# *Petrocodon gracilis* (Gesneriaceae), a New Species From Southwestern Guangxi, China

**DOI:** 10.1002/ece3.70670

**Published:** 2024-11-28

**Authors:** Tao Ding, Ming Liu, Qiang Zhang, Peng‐Fei Wang, Xing Huang, Yan‐Xiang Lin, Bo Pan, Peng‐Wei Li

**Affiliations:** ^1^ Guangxi Key Laboratory of Plant Conservation and Restoration Ecology in Karst Terrain Guangxi Institute of Botany, Guangxi Zhuang Autonomous Region and Chinese Academy of Sciences Guilin Guangxi China; ^2^ Technology Center China Tobacco Henan Industrial Co. Ltd. Zhengzhou Henan China; ^3^ Guangxi Tiandeng Topping Agriculture Co. Ltd. Chongzuo Guangxi China; ^4^ College of Pharmacy Fujian University of Traditional Chinese Medicine Fuzhou China

**Keywords:** disparity, floral morphology, Gesneriaceae, pollination, taxonomy

## Abstract

The genus *Petrocodon* is renowned for its remarkably diverse floral morphology, exhibiting a high level of disparity compared to most genera within Gesneriaceae. In this study, we present a detailed description and illustration of *Petrocodon gracilis* T. Ding & B. Pan, a new species with unique floral features that is native to Guangxi, China. This species is geographically close to *P. jingxiensis* H.S. Gao & W.B. Xu and is indistinguishable from *P. jingxiensis* in vegetative characteristics. However, it can be clearly distinguished from the latter by its shorter pedicels, slender tubular corolla, shorter corolla tube, distinctly unequal corolla lobes with upper ones linear and reflexed and lower ones oblanceolate, and included chiritoid‐like stigma. The presence of linear upper lobes and the chiritoid‐like stigma has not been previously reported in *Petrocodon,* and the emergence of this new species will further expand the morphospace occupied by this morphologically diverse genus.

## Graphical abstract

The genus Petrocodon is well known for its remarkably diverse floral morphology, displaying a high level of disparity compared to most genera within Gesneriaceae. In this study, we provide a detailed description and illustration of Petrocodon gracilis, a newly discovered species native to Guangxi, China, which further expands the morphospace occupied by Petrocodon.

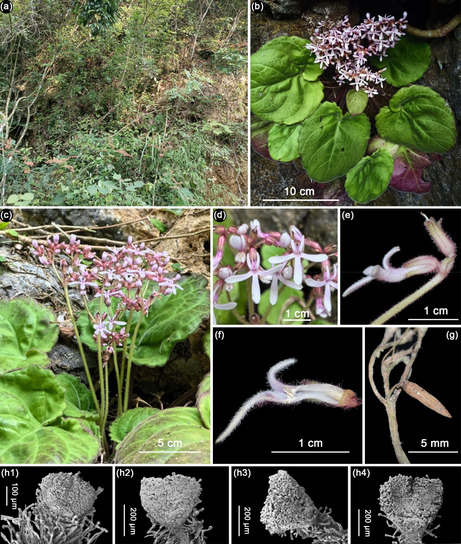

## 1 Introduction

In evolutionary biology, diversity is not just limited to the number of species present in a community or clade but also encompasses phenotypic difference among the species (Purvis and Hector 2000). Disparity is a measure of the range or significance of morphology within a group of organisms, and it focuses on the variation of traits among individuals or species within a group, highlighting differences in morphology, structure, or function (Wills, Briggs, and Fortey 1994; Oyston et al. 2016). The concept of disparity was first codified by Runnegar (1987), who was an eminent paleontologist, and popularized by Gould (1989) to refer to a variety of body plans. Floral morphology is anticipated to undergo changes over evolutionary durations and is frequently recognized as a significant catalyst for the diversification of angiosperms (Vasconcelos et al. 2018). Regardless of the criteria adopted by different taxonomists, a specific genus is expected to maintain certain morphological variations. If there is a high level of disparity, the genus tends to be divided into smaller genera. Conversely, if there are minor morphological differences compared to closely related genera, it might be merged into its allies. Nevertheless, exceptions do exist. It is not uncommon to find genera with exceptionally low or high levels of morphological disparity in angiosperms (Webster 1993; Hughes and Eastwood 2006; Marazzi and Endress 2008; Moré et al. 2015; Yu et al. 2016; Vasconcelos et al. 2019).

Gesneriaceae is a pantropical plant family with about 150 genera and over 3400 species (Weber, Clark, and Möller 2013; Ogutcen et al. 2021). The colonization of various habitats, the evolution of specialized plant–animal interactions for pollination and seed dispersal, and ancient allopolyploidization events have collectively had a profound impact on the diversification of this clade since it emerged around 70 million years ago (Roalson and Roberts 2016; Serrano‐Serrano et al. 2017; Yang et al. 2023). It has been demonstrated that certain genera within this family display a relatively consistent floral morphology, as exemplified by *Aeschynanthus* (Middleton 2007), *Columnea* (Schulte et al. 2015), *Paraboea* (Xu et al. 2008), and *Primulina* (Weber et al. 2011). In contrast, genera like *Oreocharis* (Möller et al. 2011) and *Petrocodon* exhibit a notably high degree of morphological disparity (Weber et al. 2011; Lu et al. 2017; Li et al. 2024). In particular, both the species diversity and the morphological disparity of the genus *Petrocodon* have greatly increased since its redefinition in 2011 (Wang et al. 2011; Weber et al. 2011), attributed to the ongoing description of new species in recent years, some of which exhibit a unique floral morphology (Chen et al. 2014; Yu et al. 2015; Guo et al. 2016; Lu et al. 2017; Fan et al. 2020; Zhang et al. 2020; Pan et al. 2021). Recently, four species of *Allocheilos* were reclassified and transferred to the genus *Petrocodon* (Liu et al. 2024), bringing the total number of species in this genus to 54, including one variety. The remarkable diversity in floral forms and colors within *Petrocodon* suggests a functional significance, potentially linked to pollinator shifts (Wang et al. 2010; Weber et al. 2011b; Lu et al. 2017a; Li et al. 2024). The uplift of the Qinghai–Tibet Plateau might have decreased genetic constraints on the floral architecture of *Petrocodon*, generating abundant floral morphological variants targeted by selection for ecological divergence (Li et al. 2024).

The karst area in southern China is characterized by high edaphic and topographic heterogeneity, providing a multitude of ecological niches for plant diversification (Hao, Kuang, and Kang 2015). During our field work in southeastern Guangxi, we discovered a *Petrocodon* species with a distinctive floral morphology that sets it apart from all other species within the genus. In this study, we present a comprehensive description and illustration of this newly discovered species. The introduction of this new species will further expand the morphospace occupied by *Petrocodon*.

## 2 Materials and Methods

### 2.1 Sampling, Sequencing, and Phylogenetic Reconstruction

Based on the phylogenetic results of Li et al. (2024), we collected 37 samples of *Petrocodon*, including 35 species and two individuals of the new species. Additionally, two *Primulina* species, i.e., *P. fimbrisepala* (Hand.‐Mazz.) Y.Z. Wang and *P. pinnatifida* (Hand.‐Mazz.) Y.Z. Wang, were treated as outgroups. Two leaves from different individuals of the new species were collected and immediately dried with silica gel. Total genomic DNA was extracted using a modified CTAB method (Doyle and Doyle 1987). Sequencing libraries with an insert size between 400 and 600 bp were prepared, and then genome skimming was performed on an Illumina NovaSeq6000 platform at Novogene Corporation (Tianjin, China) with 150 bp paired‐end reads. More than 3 GB of raw reads were obtained for each sample. The raw data were assessed and filtered using fastp (Chen et al. 2018), which included trimming the adaptor reads and removing the low‐quality raw reads. Clean data were used to assemble the nuclear ribosomal RNA sequences and chloroplast genomes using GetOrganelle with the recommended parameters (Jin et al. 2020). Using the assembled nuclear ribosomal sequences and chloroplast genomes as references, we mapped ITS and five chloroplast segments (*atpI‐H*, *matK*, *rps16*, *trnH‐psbA*, and *trnL‐F*) of *Petrocodon dealbatus* Hance and extracted the corresponding sequences of the two samples with Geneious v.7.1.8 (Kearse et al. 2012). We retrieved sequences for the remaining species of *Petrocodon* and the two *Primulina* species from the NCBI database. The GenBank accession number of the newly acquired sequences along with those obtained from public database are listed in Table 1. DNA sequences were aligned using MUSCLE (Edgar 2004), and adjusted manually with Bioedit (Hall 1999). Poorly aligned regions in each alignment were refined using alignmentFilter v.1.3.0 (Zhang et al. 2023) using functions maskSegment and degaps, removing sites with similarity < 50% or gaps > 50%. Phylogenetic reconstruction was performed using maximum likelihood (ML) and Bayesian methods. The ML tree was inferred using RAxML (Stamatakis 2014) with the GTRGAMMA substitution model and 1000 rapid bootstrap (BS) replicates. The Bayesian inference was conducted in MrBayes v3.2.7 (Ronquist et al. 2012). We executed a total of 10 million generations, with each of the two independent analyses utilizing four Markov Chain Monte Carlo (MCMC) chains for computation. Every 1000 generations, a single tree was randomly sampled, resulting in a total of 10,000. To ensure convergence across independent runs, we verified that the standard deviation of split frequencies remained below 0.01 and that the potential scale reduction factor approached 1 for all evaluated parameters. After eliminating the initial 25% of the trees as burn‐in, we used Tracer v1.7 (Rambaut et al. 2018) to confirm that the effective sample sizes (ESS) were greater than 200 for each parameter. Finally, we constructed a consensus tree based on a 50% majority‐rule from the remaining trees.

**TABLE 1:** Species, voucher information, and GenBank accession numbers included in this study.

Taxon	Collection no.	Location and voucher	ITS	*atpI‐H*	*matk*	*rps16*	*trnH‐psbA*	*trnL‐F*
*Pe. ainsliifolius* W.H. Chen and Y.M. Shui	LPW2013089	Napo, Guangxi, China, (PE)	MN627898	MN636930	MN637092	MN637268	MN637409	MN637548
*Pe. chongqingensis* F. Wen, B. Pan and L.Y. Su	LPW2014098	Chongqing, China, (PE)	MN627918	MN636951	MN637114	MN637290	MN637430	MN637569
*Pe. coccineus* (C.Y. Wu ex H.W. Li) Y.Z. Wang	LMT2012003	Napo, Guangxi, China, (PE)	MN627916	MN636949	MN637112	MN637288	MN637428	MN637567
*Pe. confertiflorus* H.Q. Li and Y.Q. Wang	LPW2018004	Yangshan, Guangdong, China, (PE)	MN627906	MN636939	MN637101	MN637277	MN637418	MN637556
*Pe. coriaceifolius* (Y.G. Wei) Y.G. Wei and Mich. Möller	LYQG511	Yangshuo, Guangxi, China, (IBK)	MN627901	MN636933	MN637095	MN637271	MN637412	MN637551
*Pe. dealbatus* Hance	LMT2012017	Shibing, Guizhou, China, (PE)	MN627910	MN636943	MN637106	MN637282	MN637422	MN637561
*Pe. dealbatus* var. *denticulatus* (W.T. Wang) W.T. Wang	LPW2016008	Rong'an, Guangxi, China, (PE)	MN627904	MN636936	MN637098	MN637274	MN637415	MN637554
*Pe. ferrugineus* Y.G. Wei	LPW2018013	Xincheng, Guangxi, China, (PE)	MN627900	MN636932	MN637094	MN637270	MN637411	MN637550
*Pe. gracilis* T. Ding and B. Pan (1)*	LPW2024022	Tiandeng, Guangxi, China	PQ403662	PQ431563	PQ431565	PQ399802	PQ399804	PQ399806
*Pe. gracilis* T. Ding and B. Pan (2)*	LPW2024022	Tiandeng, Guangxi, China	PQ403663	PQ431564	PQ431566	PQ399803	PQ399805	PQ399807
*Pe. hancei* (Hemsl.) A. Weber and Mich. Möller	LMT2012005	Xinning, Hunan, China, (PE)	MN627913	MN636946	MN637109	MN637285	MN637425	MN637564
*Pe. hechiensis* (Y.G. Wei, Yan Liu & F. Wen) Y.G. Wei and Mich. Möller	2013ZQ001	live plant in greenhouse, (IBK)	MN627895	MN636927	MN637089	MN637265	MN637406	MN637545
*Pe. hispidus* (W.T. Wang) A. Weber and Mich. Möller	LPW2012010	Xichou, Yunnan, China, (PE)	MN627915	MN636948	MN637111	MN637287	MN637427	MN637566
*Pe. hunanensis* X.L. Yu and Ming Li	LPW2014014	Dong'an, Hunan, China, (PE)	MN627919	MN636952	MN637115	MN637291	MN637431	MN637570
*Pe. integrifolius* (D. Fang and L. Zeng) A. Weber and Mich. Möller	LPW2013040	Longzhou, Guangxi, China, (PE)	MN627920	MN636953	MN637116	MN637292	MN637432	MN637571
*Pe. jasminiflorus* (D. Fang and W.T. Wang) A. Weber and Mich. Möller	LMT2012002	Napo, Guangxi, China, (PE)	MN627917	MN636950	MN637113	MN637289	MN637429	MN637568
*Pe. jingxiensis* (Yan Liu, H.S. Gao and W.B. Xu) A. Weber and Mich. Möller	LPW2014067	Jingxi, Guangxi, China, (PE)	MN627894	MN636926	MN637088	MN637264	MN637405	MN637544
*Pe. lancifolius* F. Wen and Y.G. Wei	LPW2018031	Huishui, Guizhou, China, (PE)	MN627902	MN636934	MN637096	MN637272	MN637413	MN637552
*Pe. laxicymosus* W.B. Xu and Yan Liu	LPW2018019	Jingxi, Guangxi, China, (PE)	MN627899	MN636931	MN637093	MN637269	MN637410	MN637549
*Pe. lithophilus* Y.M. Shui, W.H. Chen and Mich. Möller	LPW2016099	Pingbian, Guangxi, China, (PE)	MN627891	MN636923	MN637085	MN637261	MN637402	MN637541
*Pe. longgangensis* W.H. Wu and W.B. Xu	LPW2013038	Longzhou, Guangxi, China, (PE)	MN627896	MN636928	MN637090	MN637266	MN637407	MN637546
*Pe. longitubus* Cong R. Li and Yang Luo	Wangmo1073	Wangmo, Guizhou, China (PE)	MN627893	MN636925	MN637087	MN637263	MN637404	MN637543
*Pe. lui* (Yan Liu and W.B. Xu) A. Weber amd Mich. Möller	LYQG472	Jingxi, Guangxi, China, (IBK)	MN627892	MN636924	MN637086	MN637262	MN637403	MN637542
*Petrocodon luteoflorus* Lei Cai and F. Wen	LPW2014109	Libo, Guizhou, China, (PE)	MN179429	MN636937	MN637099	MN637275	MN637416	MN186392
*Pe. mollifolius* (W.T. Wang) A. Weber and Mich. Möller	LJM2012001	Zhenkang, Yunnan, China, (PE)	MN627889	MN636921	MN637083	MN637259	MN637400	MN637539
*Pe. multiflorus* F. Wen and Y.S. Jiang	LPW2018034	Cangwu, Guangxi, China, (PE)	MN627912	MN636945	MN637108	MN637284	MN637424	MN637563
*Pe. niveolanosus* (D. Fang and W.T. Wang) A. Weber and Mich. Möller	LPW2015051	Longlin, Guangxi, China, (PE)	MN627922	MN636955	MN637118	MN637294	MN637434	MN637573
*Pe. pseudocoriaceifolius* Yan Liu and W.B. Xu	LPW2018032	Huanjiang, Guangxi, China, (PE)	MN627897	MN636929	MN637091	MN637267	MN637408	MN637547
*Pe. pulchriflorus* Y.B. Lu and Q. Zhang	LPW2018018	Daxin, Guangxi, China, (PE)	MN627914	MN636947	MN637110	MN637286	MN637426	MN637565
*Pe. retroflexus* Q. Zhang and J. Guo	G43	Changshun, Guizhou, China, (IBK)	MN627890	MN636922	MN637084	MN637260	MN637401	MN637540
*Pe. scopulorum* (Chun) Y.Z. Wang	LPW2018003	Pingba, Guizhou, China, (PE)	MN627911	MN636944	MN637107	MN637283	MN637423	MN637562
*Petrocodon* sp 4	LMT2012018	Xiuwen, Guizhou, China, (PE)	MN627903	MN636935	MN637097	MN637273	MN637414	MN637553
*Petrocodon* sp 62	LPW2013135	Yangshan, Guangdong, China, (PE)	MN627907	MN636940	MN637102	MN637278	MN637419	MN637557
*Petrocodon* sp 104	LPW2018048	Zhongfang, Hunan, China, (PE)	MN627908	MN636941	MN637103	MN637279	MN637420	MN637558
*Petrocodon* sp 107	LPW2017020	Ruyuan, Guangdong, China, (PE)	MN627905	MN636938	MN637100	MN637276	MN637417	MN637555
*Pe. urceolatus* F. Wen, H.F. Cen and L.F. Fu	LPW2018029	Zhangjiajie, Hunan, China, (PE)	MN627909	MN636942	MN637104	MN637280	MN637421	MN637559
*Pe. viridescens* W.H. Chen, Mich. Möller and Y.M. Shui	85,339	Maguan, Yunnan, China, (KUN)	MN627921	MN636954	MN637117	MN637293	MN637433	MN637572
*Pr. fimbrisepala* (Hand.‐Mazz.) Y.Z. Wang	TGL0909	Jianhe, Guizhou, China, (PE)	MN627882	MN636914	MN637076	MN637252	MN637393	MN637532
*Pr. pinnatifida* (Hand.‐Mazz.) Y.Z. Wang	LPW2016072	Tianlin, Guangxi, China, (PE)	MN627846	MN636880	MN637042	MN637216	MN637357	MN637496

*Note:* Asterisks indicate sequences generated in the present study.

Abbreviations: *Pe*, *Petrocodon*; *Pr*, *Primulina*.

### 2.2 Comparative Morphological Analysis

Morphological characteristics were carefully observed for the majority of *Petrocodon* species during the extensive fieldwork conducted over the past dozen years. Additionally, colorful photos of *Petrocodon* from two regional monographs (Li and Wang 2004; Wei et al. 2010) were consulted, and the protologue of all known *Petrocodon* species was thoroughly examined. Moreover, we also scrutinized the type specimens of morphologically similar species deposited in IBK, GXMI, PE, and IBSC in person.

In order to determine the stigma type of the new species, we collected stigmas at various developmental stages and carried out scanning electron microscopy (SEM) observations. First, we gathered fresh pistils from live plants and processed them with a solution containing 70% ethanol, glacial acetic acid, and formalin (in a ratio of 18:1:1). Then, we dehydrated the fixed specimens using increasing concentrations of ethanol (70%, 80%, 90%, 100%). Subsequently, after the critical point drying, all samples were securely attached to aluminum stubs using double‐sided tape and coated with a thin layer of gold powder through a sputtering process. Finally, the stigmas were observed and photographed using the Zeiss EV018 (Oberkochen, Germany). The terminology used in the morphological description follows Harris and Harris (1994) and Weber et al. (2020).

## 3 Results and Discussion

**FIGURE 1:**
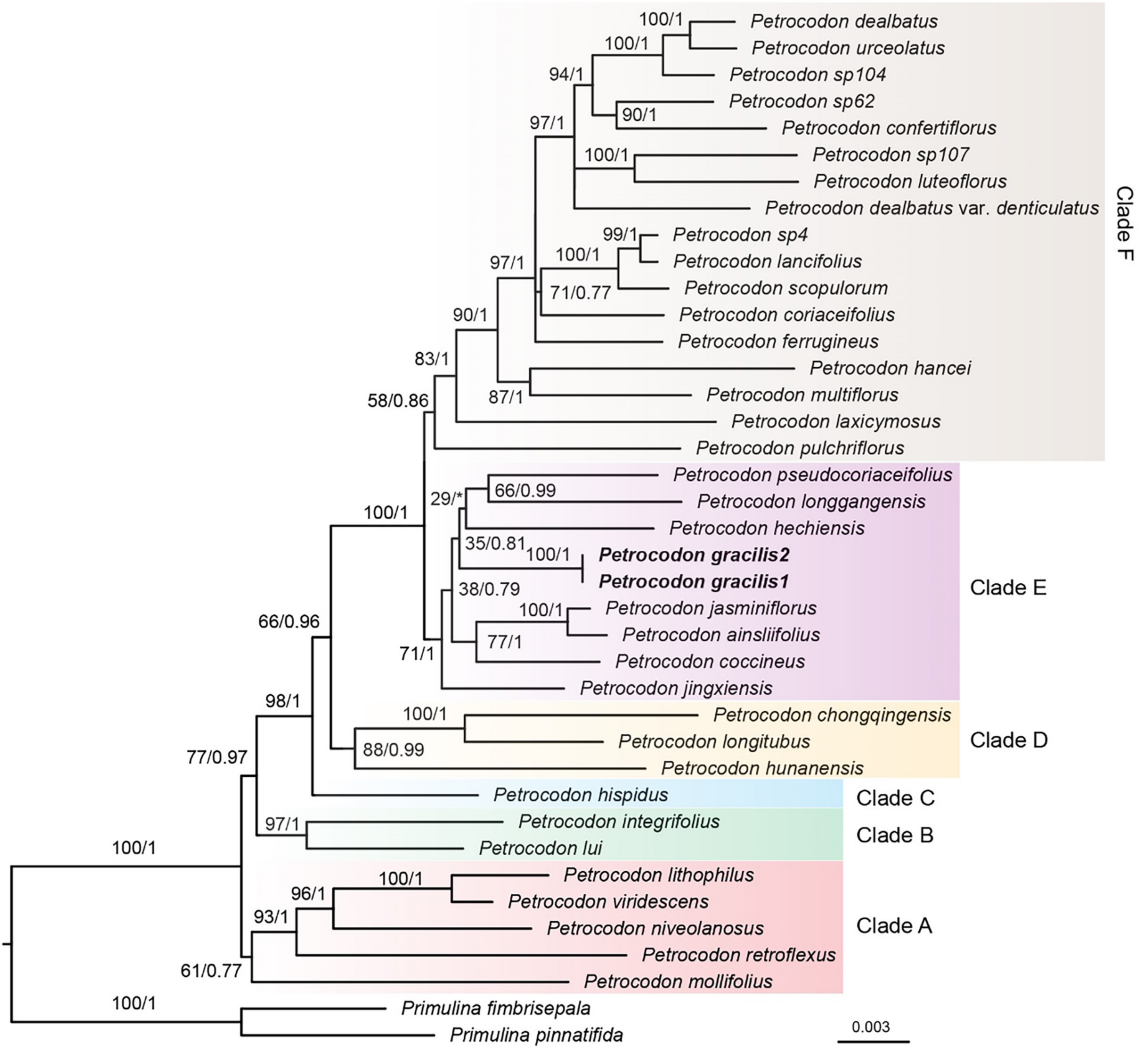
Maximum likelihood (ML) tree based on concatenated ITS and five chloroplast segments (*atpI‐H*, *matk*, *rps16*, *trnH‐psbA*, and *trnL‐F*). Numbers besides the nodes are bootstrap values from the ML method and posterior probabilities from the Bayesian inference. The tips in bold indicate the new species.

Phylogenetic analyses suggest that the genus *Petrocodon* can be divided into six clades (clades A–E, Figure 1), which is consistent with the result of Li et al. (2024). The new species is resolved in clade E and is sister to a branch consisting of *P. hechiensis* (Y.G. Wei, Yan Liu, & F. Wen) Y.G. Wei & Mich. Möller, *P. longgangensis* W.H. Wu & W.B. Xu, and *P. pseudocoriaceifolius* Yan Liu & W.B. Xu. However, there is almost no support for this relationship yet. Furthermore, other internal nodes within clade E are also poorly defined, and further exploration is needed to understand the relationships among the different species within this clade. One may relate this new species to certain *Primulina* species or *Oreocharis* species when focusing on only partial morphological features (such as the stigma type or corolla shape). Our phylogenetic results here provide unambiguous support for the generic affiliation of the new species.

**FIGURE 2:**
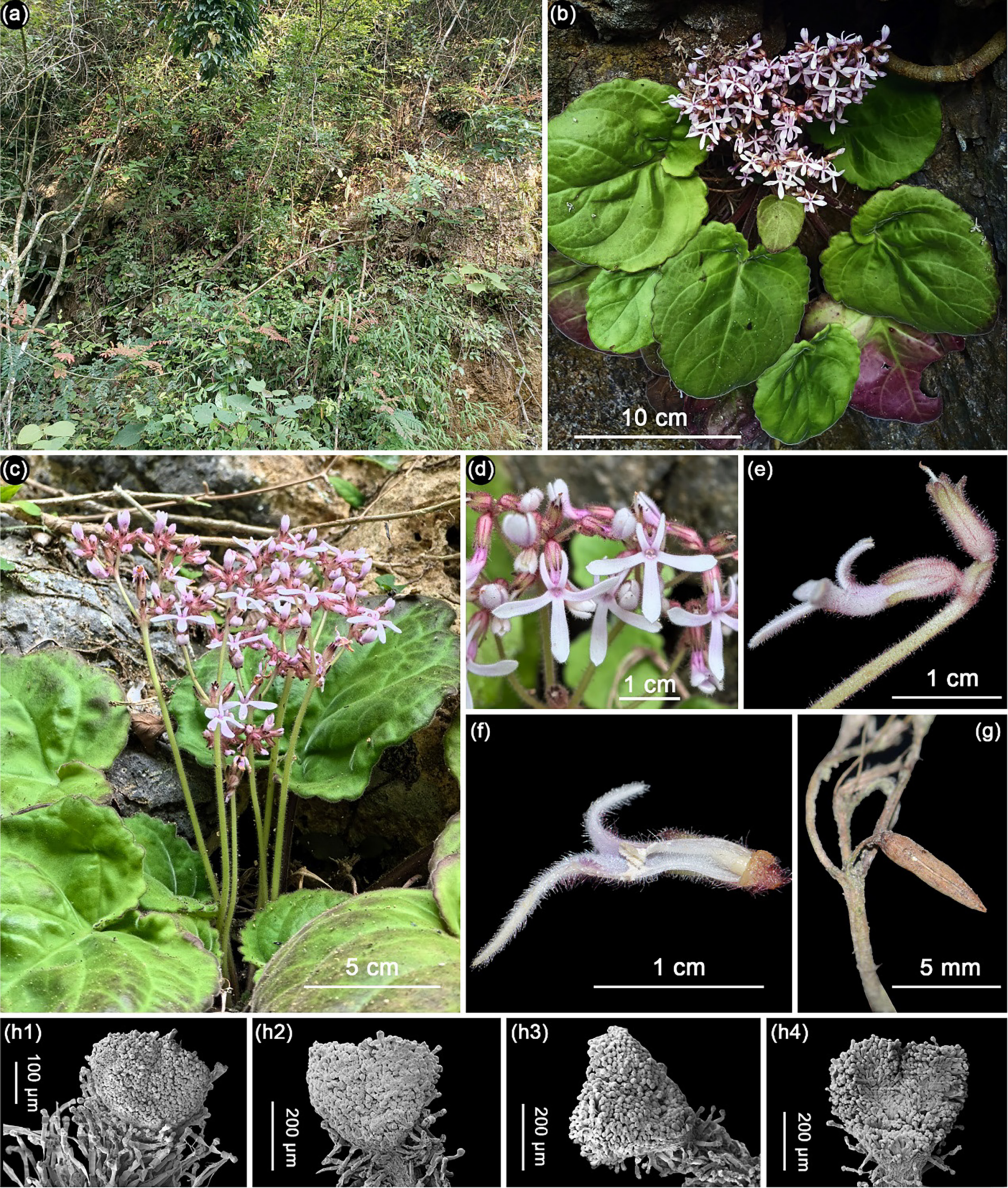
*Petrocodon gracilis*: (a) habitat, (b) habit, (c) inflorescence, (d) corolla in the front view, (e) corolla in the side view, (f) longitudinal section of the corolla, (g) capsule, (h1) stigma in a bud, and (h2–h4) stigmas in opening flowers.

In *Petrocodon*, leaves can generally be categorized into two types based on the blade shape: narrow and broad. The narrow type frequently displays leaves that are narrowly elliptic to lanceolate, a characteristic commonly observed in most species within clade F and, to a lesser extent, in clade D. The broad type frequently observed in clades A, B, C, and E can be characterized by species with blades that are usually ovate to cordate. It seems that the evolutionary trend for the leaves of *Petrocodon* is toward narrowness, as the narrower leaves mainly occur in the most derived or recent clade. There are fewer than 20 species in *Petrocodon* that show broad leaves, with the new species described in this study belonging to this category (Figure 2).

**FIGURE 3:**
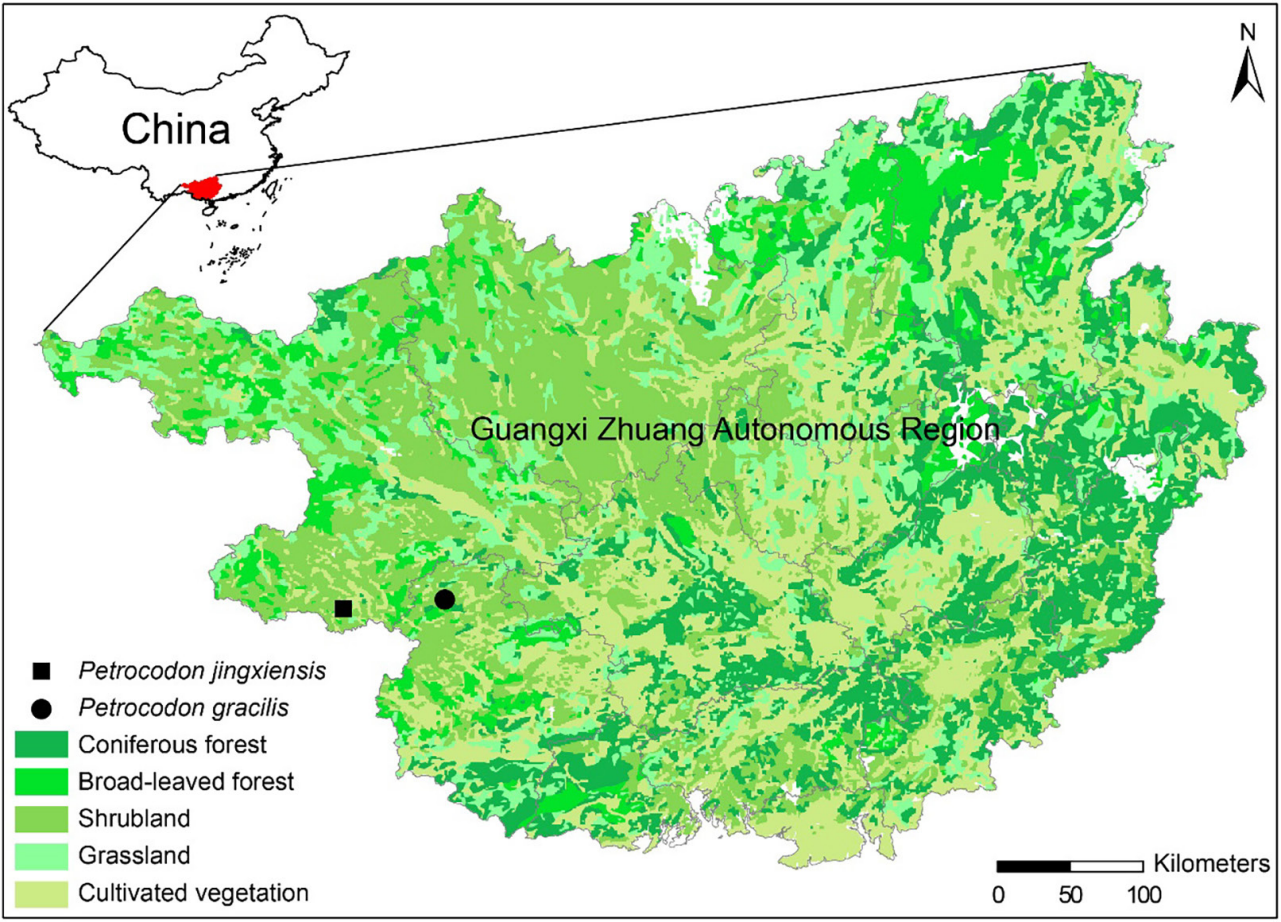
The known geographical distribution of *Petrocodon gracilis* and *Petrocodon jingxiensis*.

**FIGURE 4:**
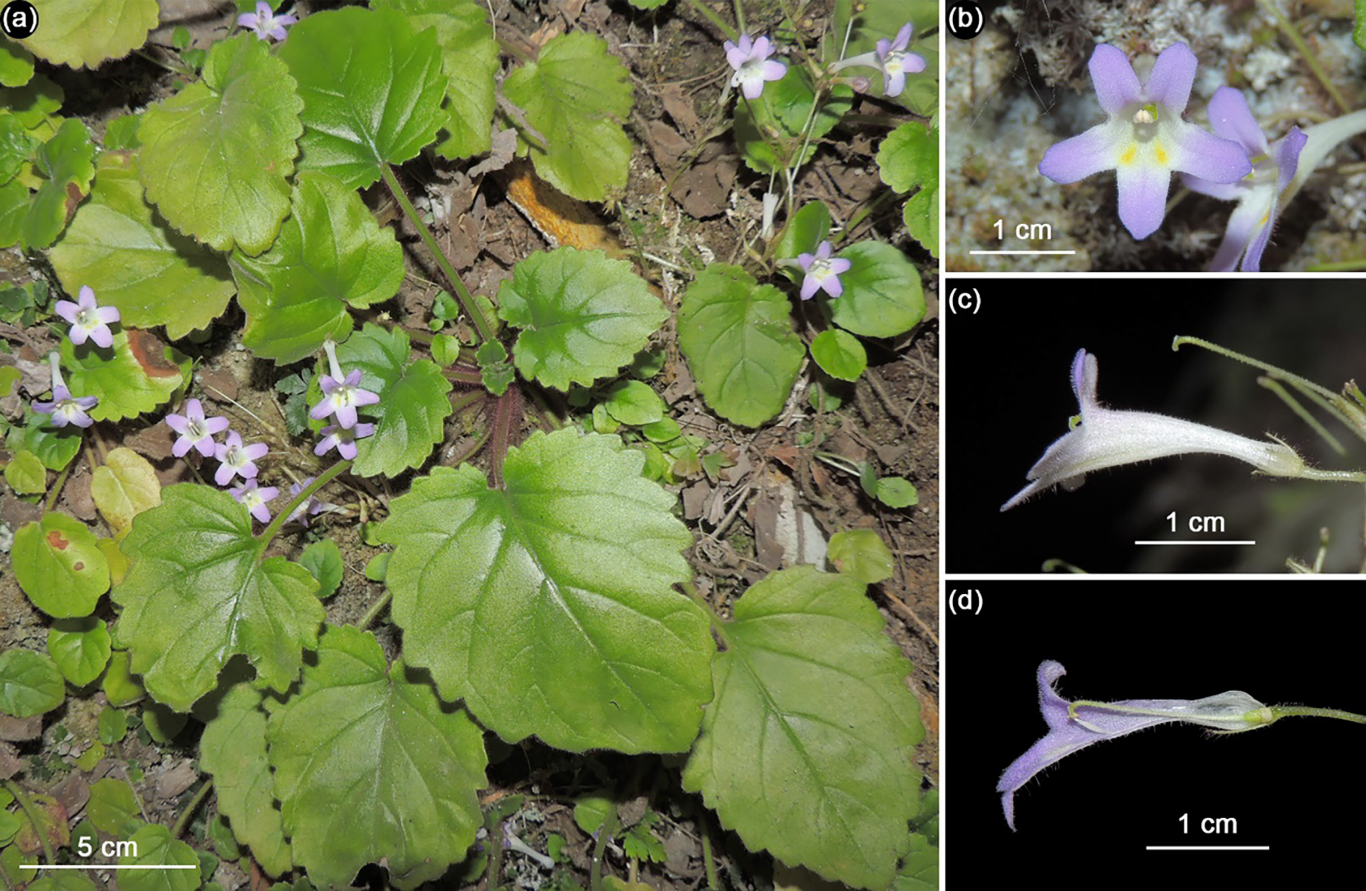
*Petrocodon jingxiensis* : (a) habit, (b) corolla in the front view, (c) corolla in the side view, and (d) longitudinal section of the corolla.

The new species is unique in its floral form, which displays linear and reflexed upper lobes and chiritoid‐like stigma (Figure 2). Actually, the nearly linear upper lobes have not been reported in *Petrocodon* before. Species with reflexed lobes can only be observed in *P. retroflexus* Q. Zhang & J. Guo, characterized by a curved‐tubular white flower with a distinctly compressed throat and a strongly retroflexed shallowly tetra‐fid upper lip (Guo et al. 2016). This contrasts with the new species, which features white flowers with pink pubescence and bifid reflexed upper lobes. Actually, the new species is unique in its floral form and is quite distinct from other known *Petrocodon* species according to our morphological observations and comparisons. However, the location of the new species is adjacent to the type locality of *P. jingxiensis* H.S. Gao & W.B. Xu (Figure 3), and they are indistinguishable when not in flower. The morphological differences between these two species mainly reside in floral features. *Petrocodon jingxiensis* is characterized by relatively long pedicels, funnel‐shaped corollas, relatively long corolla tubes, subequal ovate corolla lobes with upper ones being erect, and exserted bilobed stigmas (Figure 4). This makes it obviously different from the new species, which is characterized by shorter pedicels, narrowly tubular corollas, shorter corolla tubes, linear retroflexed upper lobes, oblanceolate lower lobes, and included chiritoid‐like stigmas (Figure 2). Detailed morphological comparisons between the two species are presented in Table 2.

**TABLE 2:** Morphological comparison between *Petrocodon gracilis* and *Petrocodon jingxiensis*.

Characters	*Petrocodon gracilis*	*Petrocodon jingxiensis*
Pedicel length	1–4 mm	10–22 mm
Corolla tube	8–12 mm, slender tubular	15–25 mm, narrowly funnel‐form
Limb color	White	Bluish violet
Upper corolla lobes	Linear and reflexed	Ovate and erect
Lower corolla lobes	Oblanceolate	Ovate to oblong‐triangular
Stigma	Included, entire and chiritoid‐like	Exserted, distinctly bilateral bilobed

In Gesneriaceae, the definition of a bilobed stigma can be ambiguous as it can refer to either upper–lower lobes or left–right lobes (Weber et al. 2020; Li et al. 2022, 2022). A chiritoid stigma is a specific type of the upper–lower stigma where only the lower lobe is developed and the apex of the lobe is usually emarginate to bifid (Weber et al. 2020). Our micromorphology observations discover that the stigma of the new species is initially left–right‐bilobed in the early stage, which then dorsal‐ventrally unequally develops into a chiritoid‐like stigma (Figure 1H1–H4). This is unusual since *Petrocodon* species are typically characterized by a capitate or left–right bilobed stigma (Wang et al. 2011; Weber et al. 2020). Perhaps, the chiritoid‐like stigma enhances the probability of successful pollination. While we may not yet understand exactly the adaptive significance of this transition related to developmental stages, the chiritoid‐like stigma indeed distinguishes the new species from all other *Petrocodon* species, making it truly unique.

Li et al. (2024) demonstrated that the morphological disparity of *Petrocodon* is rather high compared with its sister group *Primulina*, which is likely linked to their colonizing new environments and pollinator shifts following the Qinghai–Tibet Plateau uplift (Li et al. 2024). The new species in this study will further expand the morphospace occupied by *Petrocodon*. Here, it raises the question whether the genus *Petrocodon* should be split into multiple small genera given its high disparity. However, it should be noted that the current species of *Petrocodon* are derived from six oligotypic or monotypic genera (Wang et al. 2011; Weber et al. 2011). Although the redefined *Petrocodon* is not very cohesive in terms of floral morphology, it forms a group of species that have a unique evolutionary trajectory, and it is even challenging to subdivide it into subgenera. Therefore, it is evident that further splitting of the genus *Petrocodon* is not appropriate. In plant taxonomy, we tend to put more weight on the floral characters since they seem to be more conservative compared to vegetative counterparts. This may be the case for a single species, but regarding a genus, the floral characters are not necessarily stable and the vegetative ones are not always variable. In the genus *Petrocodon*, the vegetative characters are much more conserved than the floral ones, reminding us that we should put more weight on the characters related to leaves when defining this genus.

## 4 Taxonomic Treatment

### 4.1 *Petrocodon gracilis* T. Ding & B. Pan, sp. nov. Figure 2

#### 4.1.1 Diagnosis

The new species is most similar to *P. jingxiensis* but can be distinguished from the latter by the short pedicels of 1–4 mm long (vs. long pedicels 10–22 mm), a slender tubular corolla (vs. narrowly funnel‐shaped corolla), a short corolla tube of 8–12 mm long (vs. a long corolla tube of 15–25 mm long), distinctly unequal corolla lobes with the upper ones linear and reflexed and the lower ones oblanceolate (vs. subequal ovate corolla lobes with the upper ones erect), and included stigma (vs. exserted stigma).

##### 4.1.1.1 Type

China. Guangxi Zhuang Autonomous Region: Tiandeng County, Shangying Town, growing in limestone areas, under evergreen broad‐leaved forests, 23°4′5″  N, 106°59′6″  E, 600 m alt., 4 May 2024 (fl.), *T. Ding & B. Pan LPW2024022* (holotype: IBK!; isotypes: IBK! IBSC! PE!).

##### 4.1.1.2 Description

Perennial herb. Stem rhizomatous, cylindrical, unbranched, 0.5–4 (20) cm long, 0.5–1.2 cm in diameter; internodes inconspicuous. Leaves 3–9, tufted at the apex of the rhizome; petioles cylindrical, 6–13 × 0.3–0.75 cm, villous and glandular‐villous; blades broadly ovate to subround, 4.7–11 × 4.3–11.3 cm, papery when dry, both surfaces villous and glandular‐villous, round at apex, cordate at base, margins entire to serrate; lateral veins 3–4. Cymes 1–6, axillary, 1–3‐branched, 4–64‐flowered; peduncles 7.7–15.2 cm, villous and glandular‐villous; bracts 2, opposite, narrowly triangular, 3–6 × 1–1.6 mm, densely pubescent and glandular pubescent, with the entire margin. Pedicels 1–4 mm long, densely glandular and eglandular‐pubescent. Calyx 5‐parted to base; segments equal, narrowly triangular, 5–7 × 1–1.3 mm, densely villous and glandular‐villous outside, sparsely eglandular‐puberulent inside, with the entire margin and acute apex. Corolla pale pink, densely villous and glandular‐villous outside, inside nearly glabrous; tube narrowly tubular, 8–12 mm long, slightly swollen at base, 2–3 mm in diameter at the base and 3–4 mm in diameter at the mouth, throat purplish, villous, inner surface white; limb white, densely villous and glandular‐villous, distinctly 2‐lipped; adaxial lip 2‐lobed from base with lobes linear to narrowly triangular, ca 4–6 × 1 mm, reflexed; abaxial lip 3‐lobed to near base with lobes oblanceolate, ca 7–10 × 2–4 mm. Stamens 2, adnate to ca 6 mm above the abaxial side of the corolla tube base; filaments white, ca 1–1.5 mm long, glabrous; anthers coherent face to face, divaricate, ca 1.5 mm long, glabrous. Lateral staminodes 2, ca 0.4 mm long, retrorse, glabrous, adnate to 4 mm above the corolla base, adaxial staminode invisible. Disc annular, emarginate, ca 1 mm high, glabrous. Pistil 6–9.5 mm long; ovary narrowly ovoid, 2–3 mm long, densely glandular pubescent; style linear, 4–6 mm long, glandular pubescent; stigma chiritoid‐like with a lower stigma lobe lamellar with a slightly bilobed apex, ca 0.4 mm long, capsule linearly ovoid, ca 5 mm long, loculicidally dehiscing.

##### 4.1.1.3 Phenology

Flowering from April to May, fruiting from May to June.

##### 4.1.1.4 Distribution and Habitat

*Petrocodon gracilis* grows on cliffs under evergreen broad‐leaved forests in the limestone region of Tiandeng County, Guangxi, China (Figure 3).

##### 4.1.1.5 Preliminary Conservation Status

*Petrocodon gracilis* is currently known only from the type locality, where we observed approximately 200 mature individuals in total. However, field investigation is far from sufficient, and we are uncertain whether there are other populations of the new species. Following the IUCN Red List criteria (IUCN 2023), we propose to treat *P. gracilis* as data deficient.

##### 4.1.1.6 Etymology

The species epithet is derived from the almost linear upper corolla lobes. The Chinese name given is Xi Ban Shi Shan Ju Tai (细瓣石山苣苔).

## Author Contributions

**Tao Ding:** investigation (lead), methodology (lead), visualization (equal), writing – original draft (lead). **Ming Liu:** methodology (lead), visualization (equal), writing – original draft (lead). **Qiang Zhang:** writing – original draft (supporting), writing – review and editing (supporting). **Peng‐Fei Wang:** investigation (supporting), visualization (equal). **Xing Huang:** investigation (supporting), resources (lead). **Yan‐Xiang Lin:** funding acquisition (supporting), methodology (lead), writing – original draft (lead). **Bo Pan:** conceptualization (equal), funding acquisition (lead), investigation (lead), methodology (equal), writing – original draft (supporting). **Peng‐Wei Li:** conceptualization (lead), funding acquisition (lead), methodology (equal), visualization (equal), writing – original draft (lead), writing – review and editing (lead).

## Ethics Statement

The authors have nothing to report.

## Conflicts of Interest

The authors declare no conflicts of interest.

## Data Availability

The DNA sequences generated in the present study have been deposited in NCBI, and the voucher specimens of the new species were housed in IBK, IBSC, and PE.
